# Plant Growth Hormones and Micro-Tuberization in Breaking the Seed Dormancy of *Bunium persicum* (Boiss.) Fedts

**DOI:** 10.3390/plants12173163

**Published:** 2023-09-03

**Authors:** Mudasir Hafiz Khan, Niyaz Ahmad Dar, Bashir Ahmad Alie, Ghulam Hassan Mir, Uzma Fayaz, Azra Khan, Basharat Bashir, Ajaz Ahmad, Sheikh Mansoor, Yong Suk Chung, Seong Heo

**Affiliations:** 1 Advanced Research Station for Saffron and Seed Spices, Sher-e-Kashmir University of Agricultural Sciences & Technology of Kashmir, Pampore 192 121, India; aanisniyaz@gmail.com (N.A.D.); uzma78682@gmail.com (U.F.); khanazra2222@gmail.com (A.K.); mirajaz007@gmail.com (B.B.); 2 Department of Clinical Pharmacy, College of Pharmacy, King Saud University, Riyadh 11451, Saudi Arabia; ajukash@gmail.com; 3 Department of Plant Resources and Environment, Jeju National University, Jeju 63243, Republic of Korea; mansoorshafi21@gmail.com; 4 Department of Horticulture, Kongju National University, Yesan 32439, Republic of Korea; heoseong@kongju.ac.kr

**Keywords:** seedling traits, hormones, seed dormancy, micro-tuberization, *Bunium persicum*

## Abstract

*Bunium persicum* is a valuable medicinal plant with limited production but high market demand. It thrives predominantly in high-altitude regions. The main challenges hindering its widespread cultivation are seed dormancy and a lengthy seed-to-seed cycle, making its large-scale cultivation difficult. Six genotypes of *Bunium persicum* were collected from different altitudes to evaluate its germination behavior and seed dormancy. The study was conducted during 2020–23 and comprised three experiments (viz., seed germination under an open field, controlled conditions, and micro-tuberization). Under open field conditions, germination percent was genotype dependent, and the highest germination percentage, root length, and shoot length were recorded in Shalimar Kalazeera-1. Germination behavior assessment of the *Bunium persicum* revealed that treatment T_9_ (GA_3_ (25 ppm) + TDZ (9 µM/L)) is effective in breaking the dormancy of *Bunium persicum* as well as in obtaining a higher germination percent for early development of the tubers. Similarly, with regard to the effect of temperature and moisture conditions, stratification under moist chilling conditions showed effectiveness in breaking seed dormancy as the germination percentage in stratified seeds was at par with the most efficient growth hormone. With regard to the in vitro micro-propagation, direct regeneration showed multiple shoot primordia at the base of the tubers without intervening callus phase from the MS medium supplemented with BA (22.2 µM) and NAA (13.95 µM) 4 weeks after sub-culturing. Similarly, medium supplemented with JA (8.0 mg/L) and BA (22.2 µM) produced well-organized somatic embryos with shiny surfaces, which appeared at the swelled basal portion of apical stems. Further, the combination of JA (6.0 mg/L) and BA (22.2 M) was effective in developing the micro-tubers and also enhanced the weight and length of *Bunium persicum* micro-tubers.

## 1. Introduction

*Bunium persicum* ((Boiss.) Fedts.) is a low-volume high-value spice belonging to the family Umbelliferaceae. The cultivation of the crop is restricted to the high altitudes, forest areas, grassy slopes, and low-mid alpine pastoral areas of J&K [1]. It is used as a spice for culinary purposes, and the essential oil extracted from the crop has high pharmaceutical value in national and international markets [2,3,4]. Despite all the known properties of this valuable plant, there are many unknown and unexplored facts about this amazing species. Genus *Bunium* has tubers of hypocotyl or root origin. The plant reproduces naturally by sexual means through seeds, but seedlings come to flowering after four seasons of growth [5]. The demand for *Bunium persicum* is hastily increasing, while its natural habitat is shrinking because of overharvesting. The commercial cultivation of *Bunium persicum* is limited by several restraints (i.e., the crop only grows wild in scattered subpopulations; long duration of the seed-to-seed cycle, with plants reaching their first flowering after three sowing seasons; low yield and uncertain product quality; poor seed germination; and no tuber multiplication) [3]. Further, in recent years, haphazard harvesting of its seeds from wild habitats for quick economic profit has genetically eroded its ecotype and turned the plant into an endangered species. The entire crop is collected by the local natural population and sold in the market as Kalazeera or Shahi-jeera [6].

Seed germination and early crop stand are the main stages for the normal growth and development of this plant. It is a multifaceted physiological process that is influenced by various environmental factors (viz., water potential, light period, etc.). Generally, it is controlled by growth inhibitors [7]. Low seed germination is a major limitation for mass-scale cultivation of this crop. According to the reports, the main reason for the low germination rate of Umbelliferaceae species is that the embryos are small and lack oxygen during seed germination. Naturally, the seeds germinate in 90–100 days after passing through the chilling temperatures of the winter season and produce only two leaves and pinhead-sized tubers, approximately 4–6 mm, during the first year of cultivation [1,8]. The reproductive period (seed production) of *Bunium persicum* begins after 4 years and lasts up to 8–10 years as the tuber continues to grow. The economic production of the crop starts after 4 years when the tuber weight is >3 g, and this long duration from sowing of seed to flower production hinders the cultivation of this valuable crop by farmers [3]. 

Vleeshouwers et al. [9] and Warghat et al. [10] reported that stratification (chilling temperatures) plays an important role in removing the dormancy of C. carvi and *Bunium persicum*, respectively. In earlier works, induction of embryogenic callus under the influence of 2, 4-D and kinetin has been demonstrated in *Bunium persicum*, and in many other umbellifers as well [11]. Enhancement in initiation and development of micro tubers during in vitro studies of *Bunium persicum* were related to the concentrations of macro- and micro-constituents of MS medium, kinetin, and sucrose level in the medium. The use of different levels of kinetin showed a synergistic effect on growth and tuber formation, with approximately five times increase in tuber weight when sucrose concentration was increased to 60 g L^−1^. When embryo explants were used for cumin tissue culture, tremendous shoots were produced in a shorter time and without any sub-culturing [12]. Further, Tawfik and Noga [13] observed callus proliferation and stem elongation of cumin when cultures were transferred to basal medium without PGRs.

Many plant species require longer periods at low temperatures for the breaking of seed dormancy [14], while some require hot temperatures during post-harvest ripening of seed for germination when feasible conditions are available (Chauhan and Johnson, 2008). Mostly in temperate crop species, warm temperatures followed by chilling periods are required for breaking the seed dormancy, which is generally associated with morpho-physiological dormancy having underdeveloped embryos [15]. GA_3_ is a known growth hormone that plays an important role in germination, internode elongation, and flower development [16]. Various chemical treatments as well as stratification methods were used by Sharma and Sharma [17] for breaking seed dormancy, and they reported that continuous moist chilling treatments at 4 °C are effective for the release of dormancy in differentially stored and freshly harvested seeds. Furthermore, germination ceased upon shifting the seeds from 4 °C to 25 °C.

Successful cultivation of this crop species is limited by two main problems (viz., poor seed germination and long seed-to-seed cycle due to a longer dormancy period). In vitro plant regeneration of somatic embryogenesis has been performed on calli from mericarp, but very little research work has been performed on the micro-tuberization of *Bunium persicum*. Therefore, the aim of this study is to develop a protocol for breaking seed dormancy and micro-tuber development under in vitro conditions.

## 2. Results

### 2.1. Germination and Seedling Traits

**Figure 1:**
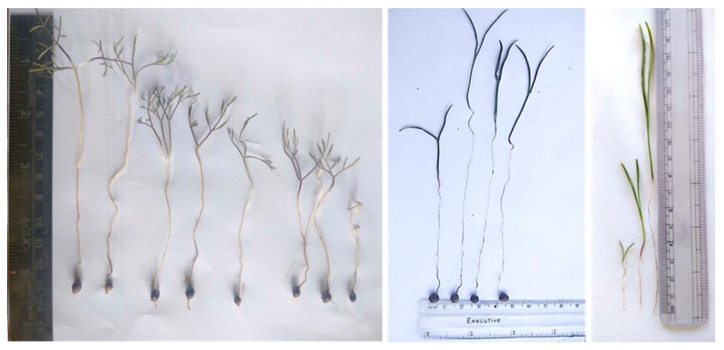
Seedling and tuber development of *Bunium persicum* under field conditions.

For the recording of observations of seedling traits, the seeds of six genotypes were sown during the 2nd fortnight of October, and the germination of seeds began during the 2nd fortnight of February. Shalimar Kalazeera-1 and Srinagar genotype showed early germination, while other genotypes germinated a few days later. The data presented in Table 1 and Figure 1 reveal that the seedling traits showed significant variation with regard to the genotypes. Among all genotypes, Shalimar Kalazeera-1 showed, significantly, the highest germination percentage (58.09%), followed by genotype (SRS/KZ/183) (46.88%) and Srinagar genotype (SRS/KZ/141) (42.80%). 

**Figure 2:**
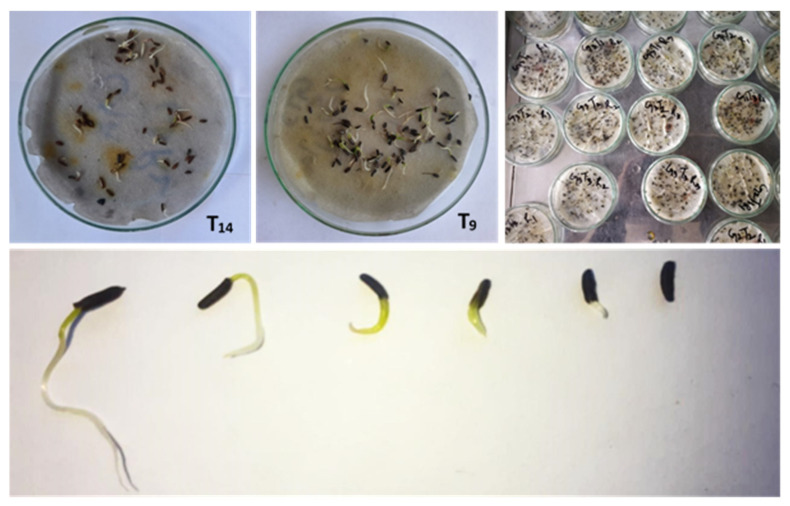
Influence of PGRs on seed germination of *Bunium persicum*.

However, the SRS/KZ/132 genotype (31.59%) showed the lowest germination percentage over the rest of the genotypes. As far as root length is concerned, Shalimar Kalazeera-1 revealed, significantly, the highest root length (10.40 cm), which was at par with the genotype (SRS/KZ/158) (9.98 cms) over other genotypes, while the lowest root length was recorded for genotype (SRS/KZ/132). A similar trend was also noticed for shoot length, with the highest and lowest values for Shalimar Kalazeera-1 and SRS/KZ/132, respectively, (Figure 1 and Figure 2). 

### 2.2. Seed Viability

For quantification of seed viability, the seed material was used from the germplasm bank of Kalazeera located at the Advanced Research Station for Saffron and Seed Spices. Freshly harvested seeds of *Bunium persicum* exhibited 89.0% viability, which was maintained at least for 10–12 months of storage. The seed viability gradually decreases with an increase in the storage period. At 22–24 months of storage, 24% decline in seed viability was noticed as compared to freshly harvested seeds.

### 2.3. Germination Behaviour Assessment

**Figure 3:**
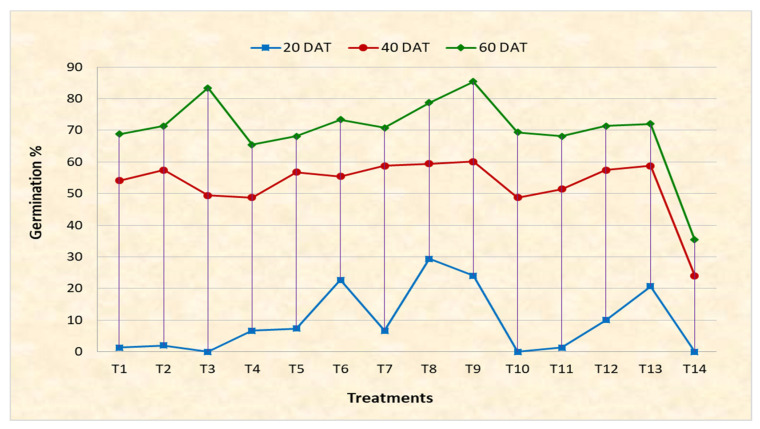
Germination behavior of *Bunium persicum* genotypes under controlled conditions.

Results of seed germination showed that stratification at a chilling temperature of 2–5 °C increased the percentage of seed germination. The combination of stratification and treatment with PGRs greatly improved the germination percentage and had a significant influence on breaking the dormancy of *Bunium persicum* seeds. A perusal of pooled data over years, presented in Table 2 and depicted in Figure 3, showed that amongst various PGRs used, the combination treatment T8 (GA_3_ (25 ppm) + TDZ (6 µM/L)) showed the highest germination percentage, which was at par with T9 (GA_3_ (25 ppm) + TDZ (9 µM/L)) at 20 days as compared to other combination treatments, as well as control; however, amongst the sole treatments, treatment T6 (TDZ (9 µM/L) exhibited a higher germination percent as compared to other treatments, while T3 (GA_3_ (50 ppm)) showed no effect on seed germination at the 20 days interval. At 40 days after treatment, the germination percent ranged between 23.0 and 70.0%. Among treatments, T9 (GA_3_ (25 ppm) + TDZ (9 µM/L)) revealed the maximum germination percent, followed by T12 (GA_3_ (25 ppm) + TDZ (3 µM/L) + jasmonic acid (50 ppm)), T7 (GA_3_ (25 ppm) + TDZ (3 µM/L)), and T8 (GA_3_ (25 ppm) + TDZ (6 µM/L)), while the lowest germination percent of 23.0 was recorded under the control treatment. 

**Figure 4:**
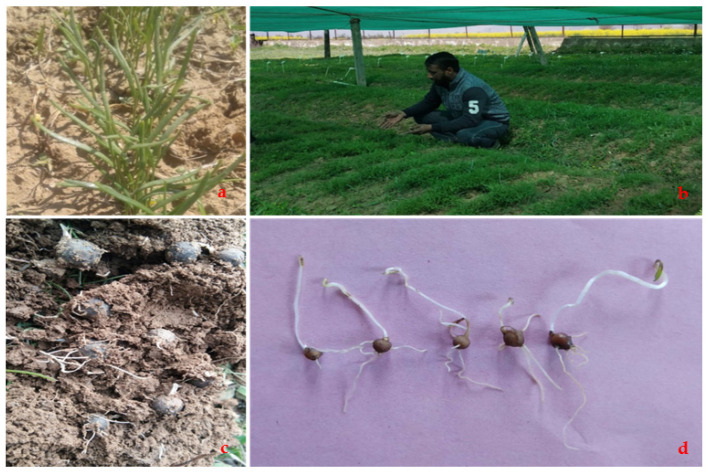
Tuber development of *Bunium persicum* under field conditions: (**a**) seed germination under pot culture; (**b**) nursery development of 14 treatments; (**c**) tuber development after 3 years; and (**d**) treatment effect on tuber development.

Data recorded at 60 days after treatment revealed the maximum germination percentage in T9 (GA_3_ (25 ppm) + TDZ (9 µM/L)), followed by T3 (GA_3_ (50 ppm)) and T8 (GA_3_ (25 ppm) + TDZ (6 µM/L)), respectively, while the lowest germination percent of 34.67 was recorded in the control (Figure 2, Figure 3 and Figure 4). Based on the observation of the two-year data, it is concluded that treatment of *Bunium persicum* seeds with T9 (GA_3_ (25 ppm) + TDZ (9 µM/L)) is effective in breaking the dormancy of the seed, as well as in obtaining a higher germination percent for early development of the tubers. 

Fourteen treatments used under lab conditions were also planted under the open field conditions during the 2nd fortnight of October 2020. Under the tested conditions, the treatments follow the same trend for the highest germination percent as was depicted previously: treatment T9 (GA_3_ (25 ppm) + TDZ (9 µM/L)) was followed by treatment T3 (GA_3_ (50 ppm)) and T8 (GA_3_ (25 ppm) + TDZ (6 µM/L)), respectively, while the lowest germination percentage was recorded for the control treatment (Figure 4). These treatments showed early germination by 25–30 days as compared to other treatments, including control. After three years, the tubers from all the treatments were uprooted, and it was noticed that the treatments T9 (GA_3_ (25 ppm) + TDZ (9 µmol/L)), T3 (GA_3_ (50 ppm)), T8 (GA_3_ (25 ppm) + TDZ (6 µM/L)) and T13 (GA_3_ (25 ppm) + TDZ (3 µM/L) + jasmonic acid (100 ppm)) produced higher and flower-bearing tubers, weighing more than 3 g as compared to other treatments (Figure 4).

### 2.4. Effects of Temperature and Moisture

Studies revealed a high impact of chilling temperature and moisture on the breaking of seed dormancy, as depicted in Table 3. Observations revealed that seeds kept under moist conditions and exposed to chilling temperature at 2–5 °C (stratification) showed the highest germination percentage as compared to dry and absence of chilling conditions. Under room temperature conditions, the dry as well as moist seeds failed to germinate and showed complete dormancy. Dry seeds at 2–5 °C showed initial germination at 40 days, and maximum germination (7.11%) was recorded after 60 days of chilling treatment. However, the seeds under moist conditions that were also exposed to chilling temperature showed germination after 20 days, and maximum germination (65.91%) was recorded after 60 days of stratification. Results of this experiment depicted that stratification along with moist conditions was responsible for breaking seed dormancy as the germination percentage in the moist-chilling seed treatment was almost at par with the most efficient growth hormone. 

Furthermore, under moist conditions, treatment of TDZ, JA, and GA_3_ exhibited significant variation in seed germination at 20, 40, and 60 days after sowing. At 20 days, TDZ showed, significantly, the highest germination (2.67%) over all other treatments, while the lowest germination (0.11%) was recorded in the control. However, GA_3_-treated seeds showed, significantly, the highest germination of 37.89% and 64.13% at 40 and 60 days of sowing, respectively, followed by jasmonic acid, with germination percentages of 11.39% and 27.67%, respectively. TDZ showed the least effect on the breaking of seed dormancy under moist chilling conditions and showed germination percentages of 12.59% and 9.76%, while the untreated control exhibited 1.21% and 3.67% germination after 40 and 60 days, respectively (Table 4).

### 2.5. In Vitro Micro-Tuberization 

**Figure 5:**
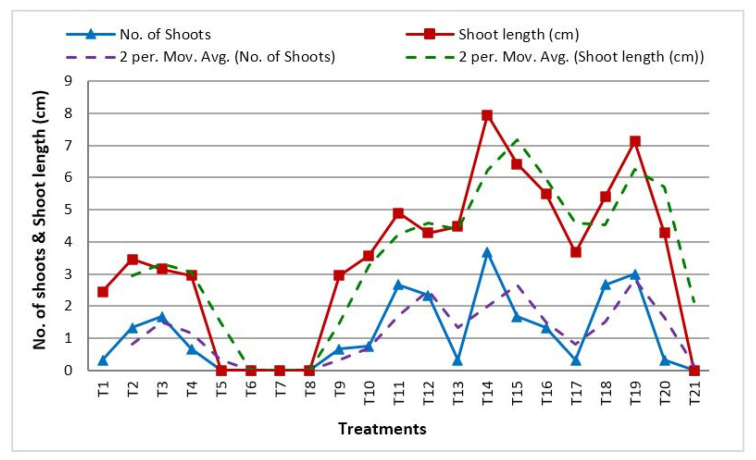
Shoot parameters as affected by PGR treatments under in vitro conditions.

The findings of the study show that after six weeks of inoculation, a hormonal combination of BA and NAA in lower concentrations was successful in promoting shoot and root multiplication in the explants over all other treatments. Among the explants used, maximum initiation was observed for tuber explants. After 4 weeks of sub-culturing, when the apical shoot with tuber was transferred to MS medium supplemented with BA (22.2 µM) and NAA (13.95 µM), multiple shoot primordia were directly regenerated from the base of the tuber and the meristematic region surrounding the apical shoot base without an intervening callus phase (Table 5, Figure 5). In MS media treated with various concentrations of JA, the highest number of multiple shoot primordia were initiated, and swelling at the base of the apical stem (meristematic area) was also seen.

**Figure 6:**
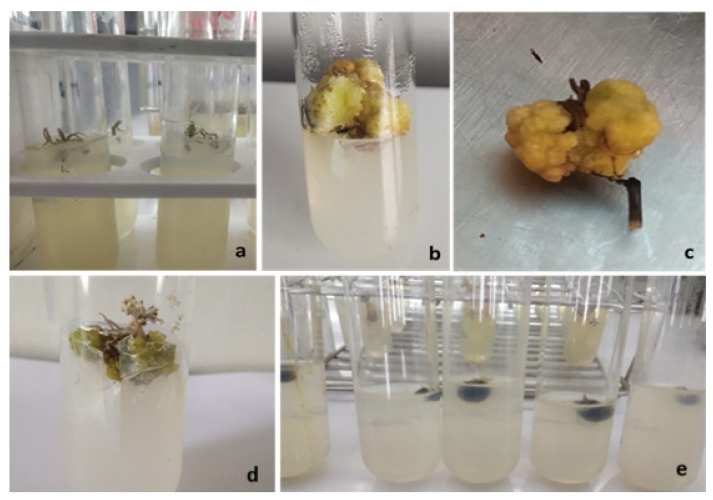
Different stages of micro-tuber development in *Bunium persicum* (**a**) green tip of apical stem; (**b**) middle portion of apical stem; (**c**,**d**) swollen basal part of apical stems; (**e**) micro-tubers.

Observations recorded after fortnight intervals revealed enlargement at the tip of the meristematic region in MS medium enriched with different concentrations of JA and NAA. Medium supplemented with JA (6.0 mg/L) and NAA (10.8 µM) revealed 88.8% response; this was followed by JA (6.0 mg/L) and BA (22.2 µM), which showed 62.5% response (Table 6). For another 3–4 weeks, the middle and tip of the apical stem remained green until turning necrotic (Figure 6a,b), and after 8 weeks of development in media supplemented with JA (8.0 mg/L) and BA (22.2 µM), well-organized, cream-colored, spherical somatic embryos with shining surfaces emerged at the swollen basal part (Bp) of apical stems without an intervening callus phase (Figure 6c,d). Amongst different combinations of hormones, the combined treatment of JA (6.0 mg/L) and BA (22.2 µM) and the sole treatment of JA (6.0 mg/L) proved efficient in micro-tuber development after 6 months of inoculation followed by combined treatment of JA (6.0 mg/L) and BA (44.4 µM) over other treatments. As far as the length and weight of *Bunium persicum* micro-tubers is concerned, MS media supplemented with a combination treatment of JA (6.0 mg/L) and BA (22.2 µM) and sole treatment of JA (6.0 mg/L) substantially enhanced the size of micro-tubers over other treatments (Figure 6e).

## 3. Discussion

In the present study, it was noticed that seed germination was genotype dependent and could not be taken as the only indicator for seed dormancy. The germination behavior of seeds is greatly affected by exposing the seeds to low temperatures of 2–5 °C, and the combination of low temperature with moisture and growth hormones increases the germination rate. Sharifi and Pouresmael [3] showed that cold stratification increased the germination rate of *Bunium persicum* and longer duration of stratification further increased the germination percentage. The combination of low temperatures with growth hormones, particularly GA_3_ and BA, showed an enhanced effect on the germination rate. The results indicate that the various processes, such as protein synthesis and respiratory systems as glycolysis, citric acid cycle, and pentose phosphate pathway, will not occur in dry seeds at room temperature, as reported by Nonogaki et al. [18], since the various physiological processes are responsible for providing the energy (adenosine triphosphate) required for germination. However, El-Dengawy et al. [19] reported that low temperatures under moist conditions trigger genes in dormant seeds, while some genes are controlled by plant hormones. Under room temperature, low germination percentage in imbibed seeds is due to the inactivation of various processes, and in fact, chilling temperature permits respiratory enzymes to retain their cell membrane structure [3]. These results are in accordance with the studies of El-Dengawy et al. [19], who reported that the effect of GA_3_ on seed germination under room temperature conditions showed that seed dormancy in *Bunium persicum* was from intermediate to low morpho-physiological dormancy. The present results are further confirmed by Hossain et al. [20], who noticed that exogenous application of GA_3_ leads to an increase in the ratio of endogenous germination promoters and cell metabolism. Therefore, the high seed germination under moist-chilling conditions pre-treated with a combination of TDZ and GA_3_ is due to an increase in the activity of endogenous phytohormones [21,22,23]. Furthermore, it is reported that the effect of TDZ on the breaking of seed dormancy is due to an increase in endogenous auxins and cytokinins of the seeds [24,25]. Bahadori and Javanbakht [26] reported that pre-treatment application of hormones causes a higher percentage of germination; however, a combination of kinetin and gibberellic acid improved the speed of germination and vigor index. The present results were also confirmed by the findings of various workers who observed the effects of stratification and chemicals on the seed germination of *Bunium persicum* [24,27]. 

In an experimental study, the effect of hormones on in vitro cultures showed that sole application of BA and NAA increased the growth rate of callus, while the combined application of BA and NAA were effective in shoot and root multiplication of the explants. The callus induction time in explants from various treatments was affected by many factors, including differences in shoot and root elongation, concentration of growth regulators added to the medium, different endogenous hormone concentrations in the explant, and differences in the cell potential, respectively. It has been reported that the interaction between auxins and cytokinins plays an important role in cell differentiation and organogenesis in tissue and other cultures [28]. Fresh weight of the callus was affected by the absorption of water and other substances in the basal environment, which is responsible for cell expansion, cell division, and new material, which leads to an increase in callus dry weight, as well as shoot and root formation [29]. The type and concentration of growth regulators, such as auxins and cytokinins, are considered as important components in callus initiation for production of secondary metabolites [30]. The present findings are well supported by the previous studies that have revealed good growth of callus by using a combination of NAA and BA in epicotyl and leaf explants of black cumin [31,32,33]. 

As noted by Campbell [34], in addition to gibberellin, there are other growth hormones such as auxin and cytokinin that affect the growth of shoots. The optimal application of auxins and cytokinins will promote cell divisions and differentiations to produce new shoots. According to Santoso [35], the difference in endogenous hormones in each explant, even when cultured in the same culture medium, can affect its response to the addition of growth regulators. The relationship between the growth regulators (exogenous) and plant endogenous substances will increase the growth and morphogenesis of the culture [24,36]. Murni [37] reported that seeds contain growth regulators such as auxins, cytokinins, gibberellins, and so on, which help in the growth and development of the seedling under in vitro as well as in vivo conditions. 

The present study revealed that jasmonic acid as sole treatment and in combination with BAA was effective in producing micro-tubers and increased the weight and length of micro-tubers in comparison with other treatments. This effect of JA on tuber formation and tuber growth has been previously reported by Pelacho and Mingo-Castel [38] while working with potato. They demonstrated that after 30 days in culture, potato tuber fresh weight was increased 6.4 times compared to kinetin-induced tuber weight. Similar to those outcomes, in *Bunium persicum*, JA may function as a chemical signal in *Bunium persicum* to initiate senescence-related processes such as tuber production, which happens after an adequate vegetative development. They further reported that this could be as a result of pleiotropic effects brought on by the influx of jasmonates from the outside, such as the stimulation or inhibition of physiological and biochemical processes in certain plant organs. These findings are also in line with the earlier reports of Jasik and Mantell [39], Cenzanoa et al. [40], Mardani et al. [41], and Gautam et al. [42], who reported that JA is effective in tuber formation under in vitro conditions. Many morphogenetic phenomena in plants, including tuberization, blooming, bulb and tuberous root formation, and so on, depend on JA and related chemicals. A thorough review has been performed on each of these topics [39,43]. Furthermore, according to some other researchers, exogenously applied JA may have a negative effect on storage organs, such as garlic bulbs [44]. Castro et al. [43] reported that JA causes plants to produce more tuberonic acid and its glucoside (TAG), which serve as signals for tuberization. According to Jasik and Mantell [39], JA, when introduced as a vapor, releases methyl jasmonates, which prevent tuberization, but when added to the medium, it accelerates micro-tuber formation. They concluded that when JA is applied solely or in combination with cytokinins in lower concentrations, it increases the young cell growth and thereby cell expansion, thus specifically inducing and increasing the weight of micro-tubers.

## 4. Materials and Methods

**Figure 7:**
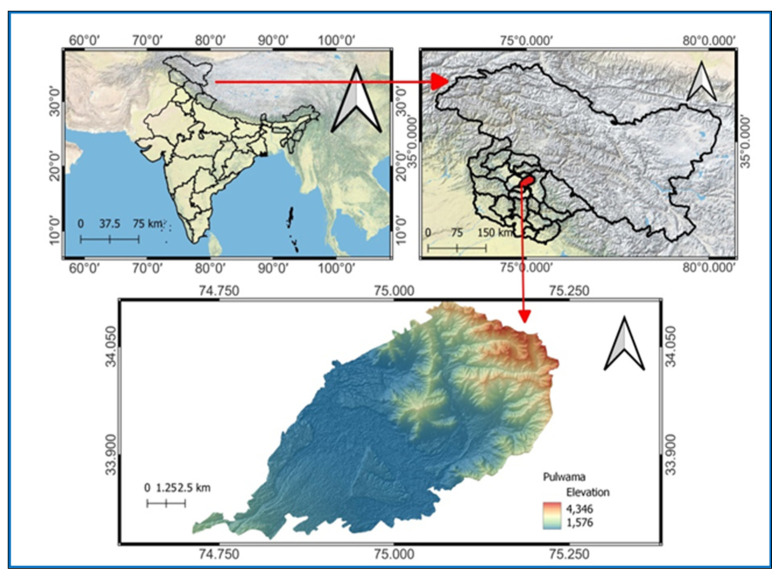
Experimental site.

The present investigation was carried out at Saffron Research, SKUAST-K, J&K, located at 74° E longitude, 34° N latitude, and approximately 1650 mts, during 2020–2023 (Figure 7). Climatically, the experimental site is in a temperate zone, characterized by cold winters and hot summers, with average annual precipitation of 812 mm, ranging between 676 to 1193 mm. The experimental material used for the present study comprises six genotypes, including Shalimar Kalazeera-1, which were collected from different altitudes with due consideration given to tuber and seed variability and participatory rural appraisal (PRA) regarding the inherent production potential of the sample. The collected planting material was planted in a randomized block design, and 20 plants were randomly selected for making observations on various seedlings and growth traits.

### 4.1. Seed Viability Determination and Surface Sterilization

For checking the viability of seeds in a short duration of time, triphenyl tetrazolium chloride (TTC) test was conducted during the storage period at regular intervals. The seeds were transversely cut into two halves and stained with TTC and then incubated for 24 h for examining staining pattern and intensity. Viable seeds, after the test, showed completely stained embryos. Surface sterilization was performed by gently washing the seeds under running tap water for 10 min, and then subjecting them to fungicide dip in hexaconazole and mancozeb. The seeds were then sterilized by shaking in 70% ethanol by placing on a magnetic stirrer for 60 s, followed by sterilization in 100 mL of 2 g/L sodium hypochlorite solution with 2 drops of Tween-20 for 15 min. The seeds were thoroughly washed three times in sterile distilled water to remove the traces of sodium hypochlorite at intervals of 10 min. After sterilization, the seeds were aseptically placed on autoclaved petri-plates lined with autoclaved filter papers, which were moistened with sterilized distilled water. The seeds were allowed to germinate by placing the petri dishes in a seed germinator at 25 ± 1 °C under continuous illumination (PAR: 40µM/m^2^/s) provided by \ fluorescent white light. Seed were treated as germinated seeds upon radicle emergence of ≥2 mm, and the germinated seeds were counted periodically.

### 4.2. Germination Behavior Assessments

Sterilized seeds were transferred into flasks and were subjected to different concentrations of GA_3_ (12, 25, and 50 ppm), TDZ (3, 6, and 9 µM/L), jasmonic acid (JA) (50 and 100 ppm), and combination treatments including GA_3_ (25 ppm) + TDZ (3 µM/L), GA_3_ (25 ppm) + TDZ (6 µM/L), GA_3_ (25 ppm) + TDZ (9 µM/L), GA_3_ 25 ppm + TDZ 3 µM/L + JA 50 ppm, and GA_3_ 25 ppm + TDZ 3 µM/L + JA 100 ppm for a duration of 24 h. Seeds dipped in sterilized distilled water served as control. After treatment, the seeds were placed in sterilized petri dishes, which were lined with sterilized filter paper with the help of sterilized forceps, at 2–5 °C for germination. The experiment was performed in a randomized complete block design with three replications for each treatment. The seeds were observed at fortnight intervals, and the numbers of germinated seeds were compared after 20, 40, and 60 days of treatment (Table 7).

### 4.3. Seed Dormancy

For breaking seed dormancy, freshly harvested seeds as well as one-year-old stored seeds were used. The seeds were first subjected to stratification at a chilling temperature of 2–5 °C, followed by dip treatments of seeds with different PGRs. 

### 4.4. In Vitro Micro-Tuberization

The trials for the development of the micro-propagation protocol of *Bunium persicum* were conducted at the Tissue Culture Lab, ARSSSS SKUAST, Kashmir, India.

Explants used: Seeds, tuber slices, the apical stem of *Bunium persicum* plants.

Sterilization: Explants were washed under running tap water for 10 min, followed by surface sterilization with 70 percent ethanol for 1 min, and 1.5% (*w*/*v*) sodium hypochlorite solution for 15 min. Before inoculation, seeds were washed repeatedly 3 times with sterilized distilled water under laminar airflow.

Media used: For tuber induction, MS media [45] supplemented with 3% sucrose and vitamins were used. The pH of the media was maintained at 5.7, which was then autoclaved at 121 °C for 15–20 min.

Hormones: Hormones used throughout the experimentation were 6-benzylaminopurine (BAP), jasmonic acid, and α-naphthalene acetic acid (NAA). Details of the PGRs used are presented in Table 8.

### 4.5. Data Analysis

Analysis of recorded data was performed as per the method suggested by Cochran and Cox [46]. Differences in mean were calculated between and among the treatments by F-test, and critical difference (C.D) at a 5% level of significance was calculated.

## 5. Conclusions

The following conclusions can be drawn from the present study. The first major finding is that stratification alone causes 65.91% seed germination after 60 days. Additionally, GA_3_ (25 ppm) + TDZ (9 µM/L) is effective in breaking the dormancy of seeds as well as obtaining a higher germination percent for the early development of tubers. Furthermore, MS media supplemented with a combination treatment of JA (6.0 mg/L) and BA (22.2 M) and sole treatment with JA (6.0 mg/L) are effective for tuber development and for increasing the weight and length of *Bunium persicum* micro-tubers.

## Figures and Tables

**Table 1:** Germination and seedling data of *Bunium persicum* genotypes under open field conditions.

Genotypes	Location	Altitude (mts)	Coordinates (Degree)	Germination (%)	Root Length (cm)	Shoot Length (cm)	Root Shoot Ratio
SRS/KZ/124	*Khrew*	1607	34.02 N, 74.93 E	39.74	8.56	9.49	1.11
SRS/KZ/132	*Budgam*	1577	33.80 N, 75.10 E	31.59	3.77	5.21	1.38
SRS/KZ/141	Srinagar	1592	34.08 N, 74.79 E	42.80	7.34	7.65	1.04
SRS/KZ/158	*Kishtwar*	1640	33.15 N, 76.09 E	35.67	9.98	10.46	1.05
SRS/KZ/183	*Gurez*	2580	34.63 N, 74.83 E	46.88	5.61	7.96	1.42
Shalimar *Kalazeera*-1	*Srinagar*	1587	34.08 N 74.83 E	58.09	10.40	11.43	1.10
**SE(m)**	**-**	**-**	**-**	**0.977**	**0.177**	**0.181**	**-**
**CD (*p* = 0.05)**	**-**	**-**	**-**	**3.120**	**0.565**	**0.586**	**-**

**Table 2:** Effect of different PGRs and chilling temperatures on germination.

Treatments	20 Days after Treatment	40 Days after Treatment	60 Days after Treatment
**T1**	1.33	54.05	68.74
**T2**	2.00	57.39	71.40
**T3**	0.00	49.38	83.41
**T4**	6.68	48.72	65.40
**T5**	7.34	56.73	68.07
**T6**	22.69	55.39	73.40
**T7**	6.68	58.73	70.74
**T8**	29.36	59.39	78.75
**T9**	24.02	60.06	85.42
**T10**	0.00	48.72	69.40
**T11**	1.33	51.38	68.07
**T12**	10.01	57.39	71.40
**T13**	20.69	58.73	72.07
**T14**	0.00	24.02	35.37
**CD (*p* = 0.05)**	1.365	5.341	7.589
**SE(m)**	0.467	1.827	2.596

**Table 3:** Effect of moisture and temperature conditions on germination.

Temperature (°C)	Moisture	Seed Germination		
		20 Days	40 Days	60 Days
15	Dry	0	0	0
20	Moist	0	0	0
2–5	Dry	0	6.19	7.11
2–5	Moist	2.90	32.00	65.91

**Table 4:** Effect of growth hormones on germination under dry and moist room conditions.

Hormones	Seed Germination		
	20 Days	40 Days	60 Days
GA_3_	0.80	37.89	64.13
JA	1.70	11.39	27.67
TDZ	2.67	9.76	12.59
Untreated	0.11	1.21	3.67
**CD (*p* = 0.05)**	0.191	2.633	4.791
**SE (m)**	0.054	0.746	0.127

**Table 5:** In vitro shoot regeneration in *Bunium persicum* from apical bud.

Treatments	NAA (µM)	Jasmonic Acid (mg/L)	BAP (µM)	Number of Shoots	Shoot Length (cm)
T1	10.8	0	0	0.33	2.45
T2	16.2	0	0	1.33	3.46
T3	21.6	0	0	1.67	3.16
T4	27.0	0	0	0.67	2.96
T5	0	2.0	0	0	0
T6	0	4.0	0	0	0
T7	0	6.0	0	0	0
T8	0	8.0	0	0	0
T9	0	0	2.22	0.67	2.96
T10	0	0	4.44	0.76	3.57
T11	0	0	22.2	2.67	4.89
T12	0	0	44.4	2.33	4.28
T13	10.8	2.0	0	0.33	4.48
T14	16.2	4.0	0	3.67	7.95
T15	21.6	6.0	0	1.67	6.42
T16	27.0	8.0	0	1.33	5.50
T17	10.8	0	2.22	0.33	3.67
T18	16.2	0	4.44	2.67	5.40
T19	21.6	0	22.2	3.00	7.13
T20	27.0	0	44.4	0.33	4.28
T21	0	0	0	0	0
CD (*p* = 0.05)	-	-	-	0.183	0.286

**Table 6:** Micro-tuber production from in vitro leaf explants.

Treatments	NAA (µM)	Jasmonic Acid (mg/L)	BAP (µM)	Swelling at Base of In Vitro Leaf (%)
T1	10.8	2.0	0	28.57
T2	16.2	4.0	0	33.33
T3	21.6	6.0	0	88.89
T4	27.0	8.0	0	33.33
T5	10.8	0	2.22	12.50
T6	16.2	0	4.44	14.28
T7	21.6	0	22.2	28.57
T8	27.0	0	44.4	50.00
T9	0	2.0	2.22	44.44
T10	0	4.0	4.44	50.00
T11	0	6.0	22.2	62.50
T12	0	8.0	44.4	28.57
CD (*p* = 0.05)	-	-	-	4.604

**Table 7:** The details of PGR treatments.

S.no	Treatments	PGR Concentration		
		GA_3_ (ppm)	TDZ (µM/L)	Jasmonic Acid (ppm)
1.	T1	12.0	0.0	0.0
2.	T2	25.0	0.0	0.0
3.	T3	50.0	0.0	0.0
4.	T4	0.0	3.0	0.0
5.	T5	0.0	6.0	0.0
6.	T6	0.0	9.0	0.0
7.	T7	25.0	3.0	0.0
8.	T8	25.0	6.0	0.0
9.	T9	25.0	9.0	0.0
10.	T10	0.0	0.0	50.0
11.	T11	0.0	0.0	100.0
12.	T12	25.0	3.0	50.0
13.	T13	25.0	3.0	100.0
14.	T14	Control (untreated)		

**Table 8:** Details of hormone treatments under in vitro conditions.

S. No	Hormones		
	BAP (µM)	NAA (µM)	Jasmonic Acid (mg/L)
1.	2.22	10.8	2.0
2.	4.44	16.2	4.0
3.	22.2	21.6	6.0
4.	44.4	27.0	8.0

## Data Availability

No data can be made available on request to the corresponding author.
